# Overexpression of *PvGF14c* from *Phyllostachys violascens* Delays Flowering Time in Transgenic *Arabidopsis*

**DOI:** 10.3389/fpls.2018.00105

**Published:** 2018-02-14

**Authors:** Bingjuan Li, Guohui Xiao, Kaisheng Luo, Zhengyi Wang, Bizeng Mao, Xinchun Lin, Xiaoqin Guo

**Affiliations:** ^1^The State Key Laboratory of Subtropical Silviculture, Zhejiang A & F University, Hangzhou, China; ^2^School of Geography and Remote Sensing, Nanjing University of Information Science and Technology, Nanjing, China; ^3^Institute of Biotechnology, College of Agriculture and Biotechnology, Zhejiang University, Hangzhou, China; ^4^Zhejiang Provincial Collaborative Innovation Center for Bamboo Resources and High-efficiency Utilization, Zhejiang A & F University, Hangzhou, China

**Keywords:** *Phyllostachys violascens*, *Pv14-3-3s*, *PvGF14c*, flowering, ectopic expression

## Abstract

14-3-3 Proteins are a family of highly conserved regulatory molecules expressed in all eukaryotic cells and regulate a diverse set of biological responses in plants. However, their functions in flowering of *Phyllostachys violascens* are poorly understood. In this study, four non-𝜀 *Pv14-3-3* genes from *P. violascens* were identified and named *PvGF14b*, *PvGF14c*, *PvGF14e*, and *PvGF14f*. qRT-PCR analyses revealed that *PvGF14b* and *PvGF14e* exhibited widely expressed in all tested bamboo tissues. *PvGF14b* was highest expression in root and lowest in immature leaf. Whereas *PvGF14c* and *PvGF14f* showed tissue-specific expression. *PvGF14c* was mainly expressed in immature and mature leaves. *PvGF14f* was highest expression in mature leaves. These four genes were not significantly differentially expressed in mature leaf before bamboo flowering and during flower development. *PvGF14b* and *PvGF14c* were not induced by circadian rhythm. PvGF14c displayed subcellular localization in the cytoplasm and PvFT in nucleus and cytoplasm. Yeast two-hybrid screening and bimolecular fluorescence complementation confirmed the interaction between PvGF14c and PvFT. The overexpression of *PvGF14b*, *PvGF14c*, and *PvGF14e* significantly delayed flowering time in transgenic *Arabidopsis* under long-day condition. These findings suggested that at least three *PvGF14* genes are involved in flowering and may act as a negative regulator of flowering by interacting with PvFT in bamboo.

## Introduction

In higher plants, flowering transition time is a major factor affecting the adaptability of plants to their environment. Flowering time in higher plants are controlled by complex signaling pathways, such as photoperiod, autonomous flowering, vernalization, and gibberellin pathways, which are independent yet interconnected with one another, eventually forming a complex network (Johanson et al., 2000; Kim et al., 2009). *SUPPRESSOR OF OVER-EXPRESSION OF CONSTANS1* (*SOC1*), *FLOWERING LOCUS T* (*FT*), and *LEAFY* (*LFY*) are considered integrating factors in these pathways (Moon et al., 2005) and are located downstream of *FLOWERING LOCUS C* (*FLC*) and *CONSTANS* (*CO*) genes, which regulate plant flowering time by integrating different flowering signals. The FT protein, which is synthesized in leaf blade and migrated along phloem tissue, plays a critical role in the shoot apical meristem (SAM). FT binds to 14-3-3 protein, and then interacts with transcription factor, *FLOWERING LOCUS D* (*FD*), thereby promoting the flowering of plants (Taoka et al., 2011). Whereas, overexpression of *OsGF14c*, an isoform of the rice 14-3-3 gene family, delayed strongly flowering time (Purwestri et al., 2009).

In eukaryotes, the 14-3-3 protein family comprises highly conserved proteins that exist as multiple isoforms in many species. Seven 14-3-3 genes are present in mammals (Arner and Holmgren, 2006), eight in rice (Chen et al., 2006), and 13 in *Arabidopsis* (Yao et al., 2007). According to homologous sequence evolution analysis, 14-3-3 proteins in plants can be divided into 𝜀 and non-𝜀 groups: the latter is plant specific (Chung et al., 1999; DeLille et al., 2001; Ferl et al., 2002). The members of the 14-3-3 family exhibit different expression patterns and appear to have varied functions. For example, *BdGF14d*, a non-𝜀 *14-3-3* gene in *Brachypodium distachyon*, can enhance salt stress tolerance (He et al., 2017). *OsGF14b*, a non-𝜀 14-3-3 protein in rice, positively regulates panicle blast resistance while negatively regulating leaf blast resistance (Liu Q. et al., 2016). Whereas, overexpression of *OsGF14c* negatively regulate flowering time in rice (Purwestri et al., 2009).

Previous studies showed that 14-3-3 proteins are involved in the regulation of flowering in many plants (Mayfield et al., 2007; Paul et al., 2008; Purwestri et al., 2009). They interact with FT in *Arabidopsis* and SP (SELF-PRUNING, ortholog of FT) in tomato (Pnueli et al., 2001; Zhang, 2012). In rice, 14-3-3 proteins act as intracellular receptors for florigen Hd3a (Heading date 3a), an ortholog of *Arabidopsis* FT (Taoka et al., 2011). Hd3a is localized in the cytoplasm and nucleus, whereas OsGF14c is mainly found in the cytoplasm. Hd3a–GF14c interaction is mainly detected in the cytoplasm, indicating that Hd3a cytoplasmic retention is increased by interacting with GF14c (Taoka et al., 2011). Thus, overexpression of *OsGF14c* plants display a delayed flowering phenotype, suggesting that OsGF14c acts as a negative regulator of flowering by interacting with Hd3a (Purwestri et al., 2009).

Similar to rice, bamboo plants belong to Gramineae but exhibit different flowering characteristics. The flowering cycle of bamboo generally lasts 3–120, and most kinds of bamboo undergoing flowering die (de Carvalho et al., 2013). However, the cause of bamboo flowering remains unclear. *Phyllostachys violascens* sprouts in early stages, produces high yields, provides good taste, offers remarkable economic benefits. As such, this species is well known for shoot production in China. *P. violascens* belong to sporadically flowering bamboos, which exhibit either a flowering with dying pattern or a flowering without dying pattern. The flowering of bamboo leading to death reduces the production of bamboo shoots and causes economic loss. Thus, many research groups tried to uncover the flowering mechanism of bamboo plants by genomes and transcriptomes (Zhang et al., 2012; Peng et al., 2013; Gao et al., 2014; Shih et al., 2014; Zhao et al., 2014). In addition, numerous genes and miRNAs related to bamboo flowering were reported (Lin et al., 2010; Gao et al., 2015; Zhao et al., 2015; Guo et al., 2016a; Liu S. et al., 2016; Zheng et al., 2017). Our group reported previously that *PvFT* played an important role in the induction of bamboo flowering (Guo et al., 2016b). However, no proteins interacting with PvFT have been identified in bamboo. In the present study, four non-𝜀 *14-3-3* genes and their expression profiles in *P. violascens* were identified. The interaction of PvGF14c and PvFT in *P. violascens* were determined. Meanwhile, overexpression of *PvGF14b*, *PvGF14c*, and *PvGF14e* was shown to delay the flowering time of transgenic *Arabidopsis* under long-day (LD) condition.

## Materials and Methods

### Plant Materials

*Phyllostachys violascens* samples were harvested from the Bamboo Garden of the Zhejiang A & F University. To detect the expression of *Pv14-3-3* during the flowering period, we collected leaf samples every 10 days from April 2 to May 31. According to our observation, the plants maintained flowering from mid-March to mid-May. The leaves from the flowering *P. violascens* were collected every 3 h for nine times to analyze the diurnal expression of the target genes. To determine the tissue-specific expression, we sampled mature leaves, immature leaves, stems, roots, and spikelets. All samples were stored at -80°C until further experiment. Wild-type (ecotype Columbia) and transgenic plants of *Arabidopsis* were cultivated in a controlled temperature room under 22°C with 16-h light/8-h dark cycle.

### Isolation of Four Pv14-3-3 Genes

Total RNA was extracted from the leaves by using the Trizol method. First-strand cDNA was synthesized using a Super Script^TM^ kit (Invitrogen, United States) according to the manufacturer’s instructions. Genomic DNA was isolated by the modified CTAB method from leaves (Guo et al., 2016b). According to *14-3-3* homologous genes of rice, BLAST was performed against *Phyllostachys heterocycla* cDNA database (Peng et al., 2013), and eight *Ph14-3-3* genes were identified. Of these genes, four candidate genes that shared more than 85% amino acid sequence identity with *OsGF14c*. Four primers used to amplify *Pv14-3-3s* were designed on the basis of four candidate genes of *P. heterocycla* (**Table 1**). The full-length cDNA and DNA sequences of four non-𝜀 *Pv14-3-3s* of *P. violascens* were amplified through PCR with the designed primers (**Table 1**). The thermocycling conditions were as follows: initialization at 94°C for 3 min; 32 cycles of denaturation at 94°C for 30 s, annealing at 50–60°C, and extension at 72°C for 50 s; and final extension at 72°C for 10 min. The amplified fragments were separated through electrophoresis on 1.5% (m/v) agarose gel and stained with ethidium bromide.

**Table 1:** Primers used in this study.

Primer	Sequences (5′–3′)	Purpose
PvGF14b1F	AACTCACCGTTGTTTACCCC	Cloning full-length DNA sequence of Pv14-3-3 genes
PvGF14b1R	CGGCTTTACTGCCCCTC	
PvGF14c1F	AAATGTCGCGGGAGGAG	
PvGF14c1R	CCATTGATTCATCCTCCCAGT	
PvGF14e1F	TCCTCCAGGTTTGATCTTC	
PvGF14e1R	TTACTGCCCATCTCCAGATT	
PvGF14f1F	GTCTAAGATCAAAGCAAAGGAAA	
PvGF14f1R	CTCAGAAATAAACATGGGTGAA	
PvGF14bF	GAGATTTGGCATTTAGAAGTTTAA	Cloning full-length cDNA sequence of Pv14-3-3 genes
PvGF14bR	GACAATAATCATAGCACAGGCA	
PvGF14cF	ACCAAAGCGAGACGACGAGA	
PvGF14cR	CAAATCCAGGGAGAACAGTATCAGT	
PvGF14eF	GGGAATCGGGAAGCCAGTA	
PvGF14eR	TGGGCACATAGACCACATTTACT	
PvGF14fF	CTCCCACGAACCCAGCAG	
PvGF14fR	GTTTTAGATAACGCAGCCCTAC	
PvGF14b2F	TAAAAATCGTAAACACCGAAG	Primer pairs used for qRT-PCR in *P. violascens*
PvGF14b2R	CTCGGAGGCAACCGTCTTGG	
PvGF14c2F	CCCCGTCCGCTTCGCCACCG	
PvGF14c2R	TTCCACATCTACCGTCTTAG	
PvGF14e2F	CGGTAACTACTAGAAACCAC	
PvGF14e2R	CTCAGAGTCAACCGTCTTAG	
PvGF14f2F	TAAGAACAAAGCAAAGGAAA	
PvGF14f2R	ACCAACATCAGCGGTCTTTA	
PvNTBF	TCTTGTTTGACACCGAAGAGGAG	
PvNTBR	AATAGCTGTCCCTGGAGGAGTTT	
PvGF14b3F	ATGTCGTCACCCGCGGAGC	Primer pairs used for qRT-PCR in transgenic plants
PvGF14b3R	GCAACCGTCTTGGCCACCTTC	
PvGF14c3F	ATGTCGCGGGAGGAGAATG	
PvGF14c3R	ATCTACTGTCTTAGCCACCTT	
PvGF14e3F	ATGTCGCAGCCTGCCGAGC	
PvGF14e3R	GTCAACCGTCTTAGCCACCTT	
PvGF14f3F	ATGTCGCAGCCTGCCGAGC	
PvGF14f3R	ATCAGCGGTCTTTACCACCTT	
AtActinF	AAAACCACTTACAGAGTTCGTTCG	
AtActinR	GTTGAACGGAAGGGATTGAGAGT	

### Bioinformatics Analysis

The open reading frames of four *Pv14-3-3s* were confirmed using the ORF Finder^1^. The exon–intron organization of four *Pv14-3-3s* was revealed using a gene structure display server^2^. The protein domains of four *Pv14-3-3s* genes were predicted using InterPro^3^. The Molecular weights and isoelectric points of 14-3-3 proteins were predicted using ExPASy program^4^. The phylogenetic tree of 14-3-3 proteins from *P. violascens*, *P. heterocycla*, *Arabidopsis*, and *Oryza sativa* was constructed using the neighbor-joining method in MEGA 5.0^5^ with bootstrap values obtained from 1000 replications.

### Expression Analysis of Four *Pv14-3-3s* Genes

Total RNAs were extracted from the collected samples by using the Trizol method. First-strand cDNA was synthesized using a Super Script^TM^ kit (Invitrogen, United States). The qRT-PCR primers were designed according to the non-conserved region of full-length cDNA sequences of four *Pv14-3-3s*. The *NTB* gene was used as the internal control gene (**Table 1**; Fan et al., 2013). The qRT-PCR analysis was performed using CFX96TM Real-Time PCR Detection System (Bio-Rad). Reactions were performed in 20-μl mixtures consisting of 10 μl 2 × SYBR Premix Ex Taq II Mix (Takara), 0.5 μl each of forward or reverse primer, 0.5 μl cDNA template (100 ng/μl), and 8 μl double distilled H_2_O. The program was 95°C for 30 s, followed by 40 cycles of 95°C for 15 s, 60°C for 30 s. The data were analyzed by the 2^-ΔΔCt^ method. Three independent replicates were performed for each experiment.

### Subcellular Localization

The full-length sequence of PvGF14c or PvFT were inserted at the C- and N-terminus of CaMV35S-GFP vector, forming PvGF14c-GFP or PvFT-GFP fusion protein, transient expression. The transient expression of the PvGF14-GFP or PvFT-GFP fusions in onion epidermal cells was made by the particle bombardment method (Wang et al., 1988). A confocal laser scanning microscope was used to observe the onion epidermal cells.

### Plasmid Construction and Transformation

The full-length ORF of *Pv14-3-3s* was attached to CaMV35S-GFP to form the plasmid *35S::Pv14-3-3s*. Afterward, the floral dipping method mediated by *Agrobacterium tumefaciens* strain GV3101 was performed to convert the plasmid into wild-type *Arabidopsis* plants. The transgenic *Arabidopsis* seeds were chosen using 50 mg/ml hygromycin in 0.5× solid medium. Positive transgenic plants were further identified through PCR. Flowering time was measured using lines with typical phenotypes. The expression level of four *Pv14-3-3s* genes in transgenic and WT *Arabidopsis* were analyzed by qRT-PCR using gene-specific primers (**Table 1**).

### Yeast Two-Hybrid (Y2H) Assay

The yeast two-hybrid (Y2H) assay was conducted following a previously reported method (Sun et al., 2015). The ORF of *PvFT* and *PvGF14c* were respectively cloned into the pGBKT7 BD vector and pGADT7 AD eukaryotic expression vectors for the swapping experiment. The Y2H was performed according to the BD Matchmaker Library Construction & Screening Kits instructions (Clontech, Palo Alto, CA, United States).

### Bimolecular Fluorescence Complementation

The ORF sequence of PvGF14c was inserted into pSAT4-cEYFP-C1-B (nYFP) vector. The ORF sequence of PvFT or AtFT was inserted into pSAT1 nEYFP - C1 (cYFP) vector, respectively. Subsequently, these vectors were introduced into DH5α. And then, the purified plasmids were introduced into the onion epidermal cells following the protocol in Section “Subcellular Localization.”

### Statistical Analysis

Statistical analysis was performed using SPSS 19.0 (SPSS Inc., Chicago, IL, United States). Differences were analyzed with one-way ANOVA followed by Tukey’s range test. Significance was accepted at the level of *P* < 0.01.

## Results

### Cloning of Four *Pv14-3-3* Genes in *P. violascens*

**FIGURE 1:**
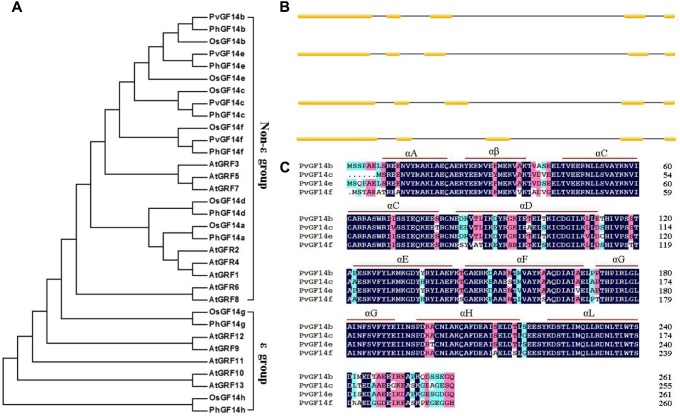
Phylogenetic and gene structure analyses of *Pv14-3-3s*. **(A)** Phylogenetic tree of *14-3-3s* from *P. violascens*, *P. heterocycla*, *O. sativa*, and *Arabidopsis* plants constructed using MAGA5.0. The registration number *14-3-3* family genes were shown in **Table 4**. **(B)** Gene structures of *Pv14-3-3s*. [scale=0.30]img001 represent the exons; [scale=0.80]img002 represent the introns. **(C)** Amino acid sequence alignments of four *Pv14-3-3s*.

We performed a BLAST 14-3-3 homologous genes of rice against *P. heterocycla* cDNA database and identified eight *Ph14-3-3* genes named *PhGF14a–h* according to the nomenclature of rice. *PhGF14a–h* showed 77, 88, 96, 83, 91, 86, 58, and 44% homolog to *OsGF14c*, respectively (**Table 2**). Phylogenetic analysis showed that *P. heterocycla* contained two 𝜀 and six non-𝜀 14-3-3 isoforms (**Figure 1A**). Four candidate genes shared more than 85% of amino acid sequence identity with *OsGF14c.* Four primers for cloning *Pv14-3-3s* were designed according to four candidate genes of *P. heterocycla*, and four *Pv14-3-3s* genes from *P. violascens* were cloned and named *PvGF14b*, *PvGF14c*, *PvGF14e*, and *PvGF14f*, respectively. All of information about four non-𝜀 Pv14-3-3 genes is shown in **Table 3**. The four Pv14-3-3 proteins contain 256–262 amino acid residues, and their molecular weight range was from 28.9 to 29.8 kDa. Their isoelectric points were from 4.70 to 4.86. The exon–intron structural analysis reveals that four non-𝜀 *Pv14-3-3* genes contain five exons and four introns (**Figure 1B**). To understand the genetic relationships of *Pv14-3-3s* in plants, we performed a phylogenetic analysis of *Arabidopsis*, *O. sativa*, *P. violascens*, and *P. heterocycla* (**Table 4**). The results indicated that *Pv14-3-3s* were closely related to *Ph14-3-3s*, as revealed by their occurrence in the same branch (**Figure 1A**), and four *Pv14-3-3s* genes belonged to the non-𝜀 group. Furthermore, we performed a multiple sequence alignment of the amino acid sequence of four *Pv14-3-3s* genes and demonstrated that four 14-3-3 proteins of *P. violascens* were highly conserved. Some sequences were completely conserved among four proteins. Eight amino acid residues in the N-terminal and 18 amino acid residues in the C-terminal exhibited large differences, suggesting that the various termini could provide different functions in each protein by interacting with a series of target proteins. Four non-𝜀 *Pv14-3-3* proteins in *P. violascens* consisted of the following nine antiparallel α-helices: αC and αD have the longest sequence of amino acids with 30–34 amino acid residues; αA–D form dimers at the interface; αC, αE, αG, and αL constitute conservative peptides with a structure similar to *14-3-3s* in other plants (**Figure 1C**).

**Table 2:** Identity between *PhGF14a–h* and *OsGF14c*.

Protein	PhGF14a	PhGF14b	PhGF14c	PhGF14d	PhGF14e	PhGF14f	PhGF14g	PhGF14h
Identity	77.00%	88.00%	96.00%	83.00%	91.00%	86.00%	58.00%	44.00%

**Table 3:** Information of four non-𝜀 *14-3-3* genes in *Phyllostachys violascens*.

Gene	ORF length (bp)	No. of introns	Deduced polypeptide		
			Length (aa)	Molecular weight (kDa)	p*I*
PvGF14b	789	4	262	29.8	4.75
PvGF14c	771	4	256	28.9	4.80
PvGF14e	789	4	262	29.7	4.70
PvGF14f	786	4	261	29.3	4.86

**Table 4:** The accession number *14-3-3* family genes in *P. heterocycla*, *O. sativa*, and *Arabidopsis*.

Genes	Amino acids	GSS
OsGF14a	264	AY224524.1
OsGF14b	262	U65956.1
OsGF14c	256	U65957.1
OsGF14d	265	U65958.1
OsGF14e	262	M276594.1
OsGF14f	260	D16140.1
OsGF14g	257	AK103145.1
OsGF14h	230	DP000010.2
AtGRFl	318	NM_001160744.1
AtGRF2	259	NM_106479.2
AtGRF3	254	NM_00103 6905.1
AtGRF4	380	NM_001198209.l_
AtGRF5	268	AY035170.1
AtGRF6	248	NM_001203346.1
AtGRF7	265	AY065274.1
AtGRF8	248	AY050996.1
AtGRF9	263	AY128764.1
AtGRFlO	254	AY081674.1
AtGRFll	255	NM_103196.3
AtGRFl 2	268	NM_102411.2
AtGRFl 3	245	AF543836.1
PhGF14a	262	FP096099
PhGF14b	263	FP101005
PhGF14c	257	100388
PhGF14d	264	FP091319
PhGF14e	233	P098170
PhGF14f	262	FP092012
PhGF14g	299	PH01002045G0220
PhGF14h	315	PH01001088G0220

### Analysis on the Expression Feature of Four non-𝜀 *Pv14-3-3s* of *P. violascens*

**FIGURE 2:**
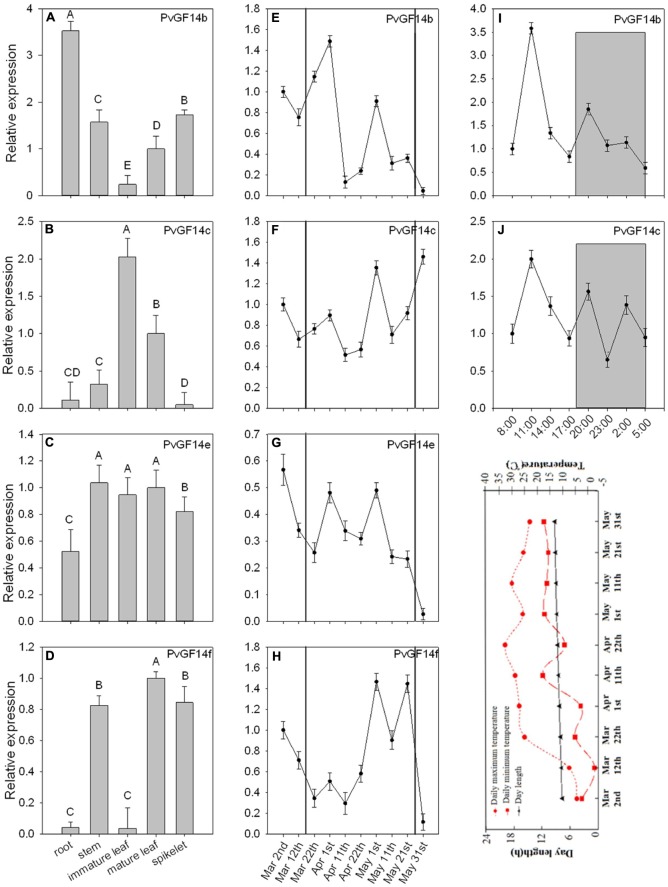
Expression analysis of four *Pv14-3-3* genes in *P. violascens*. **(A–D)** Relative expression of four *Pv14-3-3* genes in different tissues. **(E–H)** Temporal expression of four *Pv14-3-3* genes in leaves during different development stages. The period between black solid lines represents flowering time. **(I,J)** Daily expression of *PvGF14b* and *PvGF14c*. Dusk and dawn are at 17:00 and 5:30. Gray areas indicate darkness. Gray shading denotes the dark/night portion of 24 h cycle. Expression of the *PvNTB* gene was used as an internal control. Error bar indicates standard deviation. A, B indicate highly significant differences (*P* < 0.01). The image appeared below the part “**J**” is rends in daylength and temperature during the period of collecting samples.

To examine the biological functions of four non-𝜀 *Pv14-3-3s* genes, qRT-PCR was performed to analyze the temporal expression and tissue-specific expression of *14-3-3* genes of *P. violascens*. The results showed that *PvGF14b* and *PvGF14e* were constitutive expression, whereas *PvGF14c* and *PvGF14f* exhibited tissue specificity (**Figures 2A–D**). The highest expression level of *PvGF14f* was observed in the stem. Its expression was undetectable in the immature leaves and in the roots (**Figure 2D**). The highest expression level of *PvGF14c* was found in the leaves. Conversely, a low expression level was recorded in the root, stem, and spikelet (**Figure 2B**). The samples of mature leaves collected were used to detect the expression levels of four *Pv14-3-3s* genes before bamboo flowering and during flowering development. The expression levels of each *Pv14-3-3s* genes did not significantly different between these two development stages (**Figures 2E–H**). Before bamboo flowering, the relative expression level of three *Pv14-3-3s* genes, *PvGF14b*, *PvGF14c*, and *PvGF14f* were similar and higher than it of *PvGF14e.* Furthermore, the data showed that *PvGF14b* and *PvGF14c* were expressed in 24 h with highest at 11 am, but without obvious difference between day and night, suggesting that *PvGF14b* and *PvGF14c* were not shown to have diurnal rhythm expression (**Figures 2I,J**).

### The Influence of Ectopic Expression of Four Pv14-3-3 Genes on Flowering Time in Transgenic *Arabidopsis*

**FIGURE 3:**
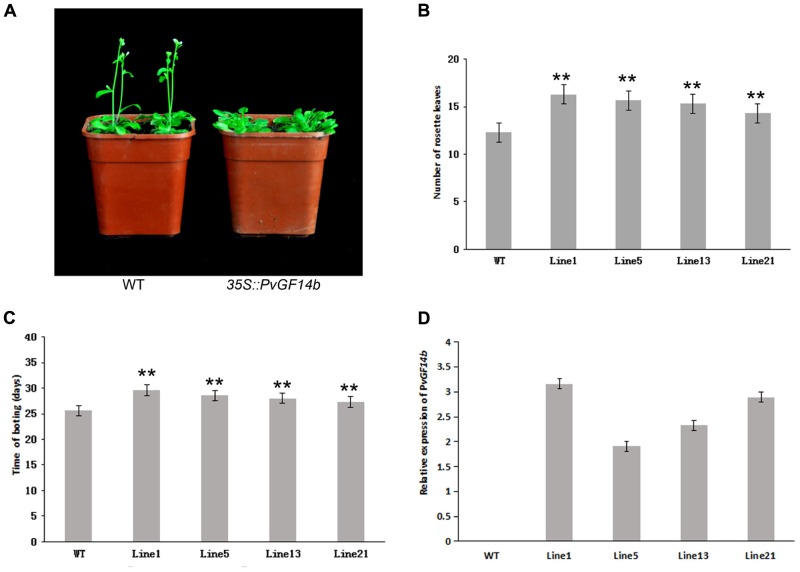
Phenotype analysis of *35S::PvGF14b Arabidopsis* plants under LD conditions. **(A)** Phenotype differences in flowering between *35S::PvGF14b* transgenic plants and wild-type plants. Photographs were taken at 26-day-old wild-type plants. **(B)** The number of rosette leaves in T2 *35S::PvGF14b* in transgenic plants (*n* = 20) and wild-type plants. **(C)** Bolting time in T2 *35S::PvGF14b* transgenic plants (*n* = 20) and wild-type plants. **(D)** qRT-PCR expression analysis of *PvGF14b*. Asterisks “^∗∗^” indicate significant differences in comparison with the WT at *P* < 0.01.

**FIGURE 4:**
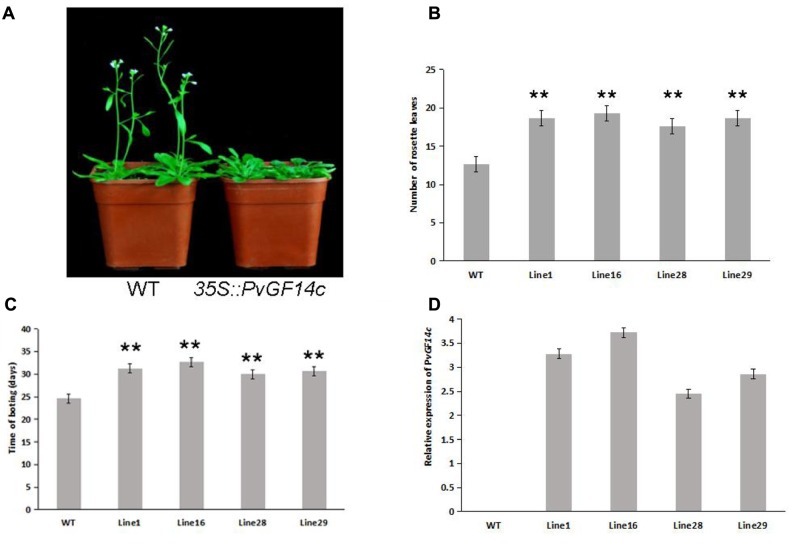
Phenotype analysis of *35S::PvGF14c Arabidopsis* plants under LD conditions. **(A)** Phenotype differences in flowering between *35S::PvGF14c* transgenic plants and wild-type plants. Photographs were taken at 28-day-old wild-type plants. **(B)** The number of rosette leaves in T3 *35S::PvGF14c* in transgenic plants (*n* = 24) and wild-type plants. **(C)** Bolting time in T3 *35S::PvGF14c* transgenic plants (*n* = 24) and wild-type plants. **(D)** qRT-PCR expression analysis of *PvGF14c*. Asterisks “^∗∗^” indicate significant differences in comparison with the WT at *P* < 0.01.

**FIGURE 5:**
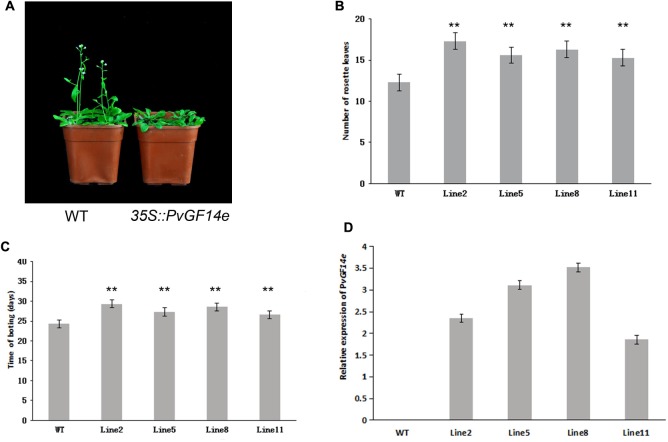
Phenotype analysis of *35S::PvGF14e Arabidopsis* plants under LD conditions. **(A)** Phenotype differences in flowering between *35S::PvGF14e* transgenic plants and wild-type plants. Photographs were taken at 25-day-old wild-type plants. **(B)** The number of rosette leaves in T2 *35S::PvGF14e* in transgenic plants (*n* = 20) and wild-type plants. **(C)** Bolting time in T2 *35S::PvGF14e* transgenic plants (*n* = 20) and wild-type plants. **(D)** qRT-PCR expression analysis of *PvGF14e*. Asterisks “^∗∗^” indicate significant differences in comparison with the WT at *P* < 0.01.

**FIGURE 6:**
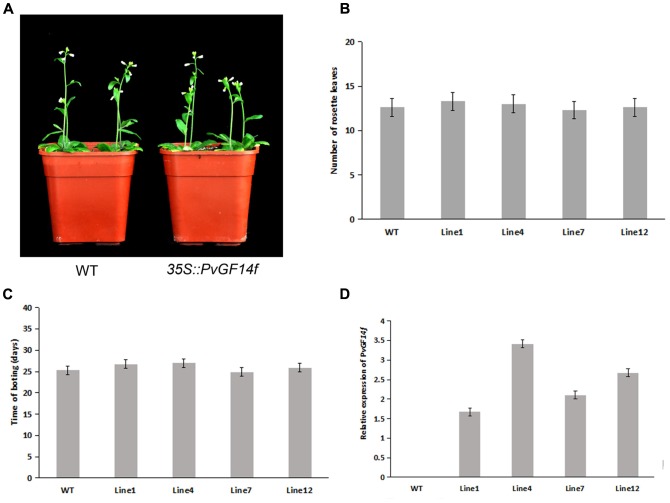
Phenotype analysis of *35S::PvGF14f Arabidopsis* plants under LD conditions. **(A)** Phenotype differences in flowering between *35S::PvGF14f* transgenic plants and wild-type plants. Photographs were taken at 27-day-old wild-type plants. **(B)** The number of rosette leaves in T2 *35S::PvGF14f* in transgenic plants (*n* = 20) and wild-type plants. **(C)** Bolting time in T2 *35S::PvGF14f* transgenic plants (*n* = 20) and wild-type plants. **(D)** qRT-PCR expression analysis of *PvGF14f*.

To demonstrate whether four 14-3-3 genes can influence flowering in plants, we constructed the overexpression vectors 35S::*PvGF14b*, 35S::*PvGF14c*, 35S::*PvGF14e*, and 35S::*PvGF14f* which were transformed to *Arabidopsis*, respectively, and got 31 T1 *GF14b*-ox plants, 42 T1 *GF14c*-ox plants, 17 T1 *GF14e*-ox plants, and 15 T1 *GF14f*-ox plants. At last, four independent lines of *GF14b*-ox, *GF14e*-ox, and *GF14f*-ox in the T2 generation, as well as four independent lines of *GF14c*-ox in the T3 generation were grown under greenhouse condition for further analysis. 35S::*PvGF14b*, 35S::*PvGF14c* and 35S::*PvGF14e* delayed flowering in transgenic *Arabidopsis* (**Figures 3**–**5**). Whereas, *PvGF14f*-overexpressing plants showed the similar flowering time as that of the wild-type (**Figure 6**). The number of rosette leaves of *PvGF14b*, *PvGF14c*, and *PvGF14e* transgenic plants was 2–5, 5–7, and 3–6 more than that of the wild-type, respectively (**Figures 3**–**5**). The bolting time in 35S::*PvGF14b*, 35S::*PvGF14c* and 35S::*PvGF14e* transgenic *Arabidopsis* plants was 3–4, 6–8, and 4–6 days later than that in the wild-type (**Figures 3**–**5**).

To determine whether the flowering time in transgenic *Arabidopsis* had relation with the expression of examined genes, we detected the transcript levels of *PvGF14b*, *PvGF14e*, and *PvGF14f* in T2 lines of 35S::*PvGF14b*, 35S::*PvGF14e*, and 35S::*PvGF14f Arabidopsis*, as well as *PvGF14c* expression level in T3 lines of 35S::*PvGF14c Arabidopsis*, respectively. The data revealed that expression levels of examined genes in different transgenic lines were significantly increased (**Figures 3**–**6**). The flowering time was positively associated with the expression of *PvGF14c* in transgenic *Arabidopsis* plants (**Figure 3D**). However, there was no correlation between other three *PvGF14s* mRNA abundance and the flowering time (**Figures 4D**, **5D**, **6D**).

### Subcellular Localization of PvGF14c

**FIGURE 7:**
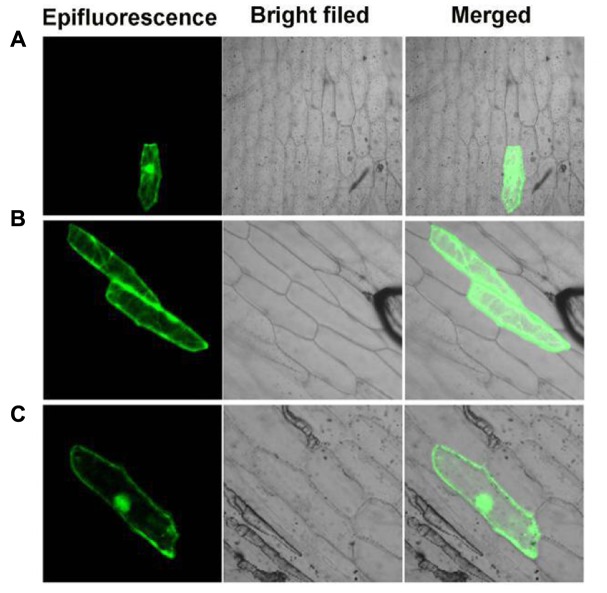
Subcellular localization of PvGF14c and PvFT protein. **(A)** Cells bombarded with 35S::GFP. **(B)** Cells bombarded with PvGF14c-GFP. **(C)** Cells bombarded with PvFT-GFP.

Previous studies showed that OsGF14c predominantly localized in the cytoplasm and OsGF14c-ox plants were delayed by 5–20 days in flowering relative to wild-type plants (Purwestri et al., 2009). In addition, based on the data above, we found that the effect of *PvGF14c* on flowering time of transgenic plants was stronger than other three *PvGF14s* genes (**Figures 3**–**6**) and phylogenetic tree showed that PvGF14c was closest related to OsGF14c (**Figure 1**). Therefore, we built PvGF14c-GFP fusion constructs driven by CaMV35S promoter to analyze the intracellular localization of PvGF14c and to investigate the molecular mechanism of PvGF14c. These constructs were introduced into onion epidermal cells for transient expression. As expected, the empty GFP signals were ubiquitously distributed throughout the cells (**Figure 7A**). PvGF14c-GFP fusion proteins were localized in the cytoplasm (**Figure 7B**).

### Interaction between PvGF14c and PvFT Proteins

**FIGURE 8:**
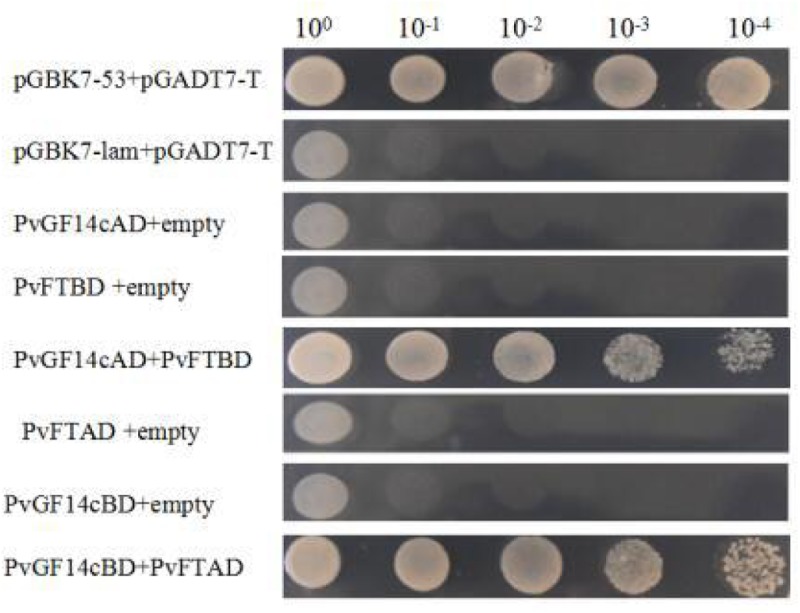
Identification of the interaction between PvGF14c protein and PvFT protein in yeast. The interaction between pGBKT7-53 and pGADT7-T was the positive protein–protein control, whereas the interaction between pGBKT7-lam and pGADT7-T was the negative protein–protein control.

**FIGURE 9:**
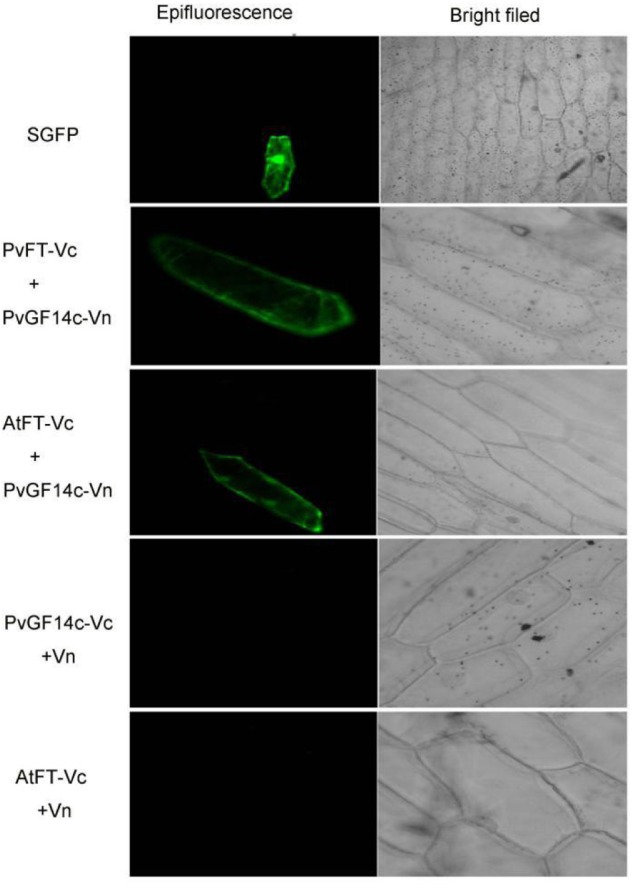
*In vivo* interaction between PvGF14c and PvFT or AtFT in onion epidermal cells by BiFC.

Previous presentation showed that the binding of 14-3-3 to Hd3a is important for flowering and OsGF14c interacted with Hd3a by Y2H screen and bimolecular fluorescence complementation (BiFC; Purwestri et al., 2009; Taoka et al., 2011). Thus, Y2H screening was performed to identify the relationship between PvGF14c and PvFT. The results demonstrated that PvGF14c could interact with PvFT protein as either a bait or a prey in *P. violascens* (**Figure 8**). Meanwhile, we used BiFC to determine the interaction of PvFT and PvGF14c in onion cell. As shown in **Figure 9**, the PvFT and PvGF14c interaction was detected in the cytoplasm. However, PvFT alone was detected in cytoplasm and nucleus (**Figure 7C**).

### The PvGF14c and AtFT Complex Is Localized in the Cytoplasm

Purwestri et al. (2009) presented that the interaction of Hd3a and OsGF14c resulted in Hd3a retaining in cytoplasmic and delayed flowering time in OsGF14c-ox. Our data showed that *PvGF14c*-ox plants also displayed the delayed flowering phenotype. Therefore, we determined the distribution of PvGF14c and AtFT *in vivo* by using BiFC. As shown in **Figure 9, the PvGF14c and AtFT** complex was located in cytoplasm.

## Discussion

Plant 14-3-3 proteins act in a phosphorylation network and play an important role in plant growth and development. 14-3-3 Proteins interact with FT in *Arabidopsis*, with SP in tomato, and Hd3a in rice (Pnueli et al., 2001; Purwestri et al., 2009; Zhang, 2012). Studies on overexpression transgenic plants and mutant plants have revealed that OsGF14c functions as a negative regulator of flowering (Purwestri et al., 2009). However, the exact role of 14-3-3 proteins in the regulation of flowering in *P. violascens* has not yet to be reported.

In this study, four *14-3-3* genes, which share more than 85% amino acid sequence identity with *OsGF14c*, were identified in *P. heterocycla* and synthesized from *P. violascens*. The molecular masses and isoelectric points of four Pv14-3-3 proteins were consistent with the values of 14-3-3 families in reported plants (Xu and Shi, 2006; Li and Dhaubhadel, 2011; Qin et al., 2016). In *Arabidopsis*, the non-𝜀 group comprises four exons and three introns, introns insert position quite conservative (Wu et al., 1997). By comparison, the 𝜀 group generally includes six to seven exons, and the genetic structures of the 𝜀 group are different from those of the non-𝜀 group (DeLille et al., 2001). But the exon–intron structure analysis revealed that the four *Pv14-3-3s* consisted of five exons and four introns (**Figure 1B**), which are similar to non-𝜀 *14-3-3s* in rice (Chen et al., 2006).

Phylogenetic analysis indicated that four Pv14-3-3s proteins all belonged to the non-𝜀 group. *Pv14-3-3s* were closely related to *Ph14-3-3s*, and they gathered in the same branch. Meanwhile, *Pv14-3-3s* were closely related to non-𝜀 *14-3-3s* of *O. sativa.* The conservation of non-𝜀 *14-3-3s* between *P. violascens* and *O. sativa* indicated that the functions of four non-𝜀 *Pv14-3-3s* may be similar to those of non-𝜀 14-3-3s in *O. sativa*. Especially, PvGF14c is the highest identity with the OsGF14c which act as intercellular receptor for florigen, and OsGF14c-overexpressing plants display a delay flowering phenotype (Purwestri et al., 2009; Taoka et al., 2011). Thus, the regulation of flowering by *Pv14-3-3s* in *P. violascens* should be further investigated.

To examine the biological functions of four *Pv14-3-3s*, we conducted temporal-spatial expression analysis and observed that the expression pattern of these four genes were diverse, and there were various degree of expression in leaf. The mRNA of PvGF14c accumulated strongest in leaf relative to other tissues (**Figure 2B**). In addition, their expression levels did not differ before bamboo flowering and during flowering development (**Figures 2E–H**). The expression of *PvGF14c* and *PvGF14b* showed no diurnal changes, suggesting that the expression of these two genes were independent of photoperiod during plant development (**Figures 2I,J**).

In rice, GF14c-overexpressing plants displayed late flowering time and the knockout mutants exhibited early flowering under short-day conditions (Purwestri et al., 2009). In our study, *PvGF14c*-overexpressing *Arabidopsis* plants underwent late flowering under LD conditions, which is similar to *OsGF14c* delaying strongly flowering time (Purwestri et al., 2009). Considering that the 14-3-3 protein family were highly conserved, we also determine whether three other 14-3-3 proteins could regulate flowering. The results showed that *PvGF14b* and *PvGF14e* overexpression delayed slightly flowering time in transgenic *Arabidopsis*. Whereas, *PvGF14f*-ox plants showed the similar flowering time as that of the wild-type (**Figures 3**–**6**).

14-3-3 Isoforms bind to many kinds of proteins including regulators of flowering time, such as CO (Mayfield et al., 2007), and florigen FT (Pnueli et al., 2001). In rice, 14-3-3 proteins act as intercellular receptors for Hd3a which is localized in the cytoplasm and nucleus, whereas GF14c are mainly found in the cytoplasm. Hd3a–GF14b and Hd3a–GF14c interactions were detected mainly in the cytoplasm (Taoka et al., 2011). In wild-type of rice, Hd3a protein transported from leaves to the SAM is first received by OsGF14c protein in the cytoplasm, and then Hd3a–14-3-3 complex is translocated to the nucleus, where it forms a larger protein complex, named florigen activation complex, with OsFD1, then induces transcription of *OsMADS15*, leading to flowering (Taoka et al., 2011, 2013). 14-3-3 Proteins regulate their interaction proteins through a variety of mechanisms such as altering their subcellular localization (Roberts, 2003). The delayed flowering phenotype in GF14c-ox plants can be explained by the increased cytoplasmic retention of Hd3a by OsGF14c which are highly expressed in plants (Purwestri et al., 2009; Taoka et al., 2011). In our study, PvGF14s-ox plants exhibit delayed flowering. The interaction of PvGF14c and PvFT in *P. violascens* as well as PvGF14c and AtFT complex locating in cytoplasm were determined. These experiments hinted that high expression of PvGF14c gene regulate the import of FT into the nucleus in transgenic *Arabidopsis*. The floral induction could be attenuated by the cytoplasmic retention of FT by PvGF14s.

In flowering regulation, whether Pv14-3-3 proteins bind to PvFT and PvFD, forming a complex, and participate in the molecular machinery of flowering regulation is unknown, and the molecular mechanism of *PvGF14-3-3s* in regulating flowering should be further investigated.

## Author Contributions

XG, BL, and GX conceived and designed the experiments. BL, GX, ZW, BM, and XL conducted and analyzed the experiments. KL helped to analyze the data. BL and XG wrote the paper. All authors read and approved the manuscript.

## Conflict of Interest Statement

The authors declare that the research was conducted in the absence of any commercial or financial relationships that could be construed as a potential conflict of interest.

## Footnotes

1 http://www.ncbi.nlm.nih.gov/gorf

2 http://gsds.cbi.pku.edu.cn

3 http://www.ebi.ac.uk/

4 http://web.expasy.org/compute_pi

5 www.megasoftware.net
